# Fixed partial dentures in an up to 8-year follow-up

**DOI:** 10.1590/S1678-77572010000400008

**Published:** 2010

**Authors:** Maximiliano Sérgio CENCI, Paulo Antônio da Rosa RODOLPHO, Tatiana PEREIRA-CENCI, Altair Antoninha Del Bel CURY, Flávio Fernando DEMARCO

**Affiliations:** 1 DDS, MSc, PhD, Adjunct Professor, Department of Operative Dentistry, Dental School, Federal University of Pelotas, Pelotas, RS, Brazil.; 2 DDS, Private Dental Practitioner, Caxias do Sul, RS, Brazil.; 3 DDS, MSc, PhD, Professor, Piracicaba Dental School, State University of Campinas, Piracicaba, SP, Brazil.; 4 DDS, PhD, Associate Professor, Department of Operative Dentistry, Dental School, Federal University of Pelotas, Pelotas, RS, Brazil.

**Keywords:** Survival rate, Composite resins, Fixed partial denture, Fiber-reinforced composites

## Abstract

Information on the longevity of fiber-reinforced composite fixed partial dentures (inlay FPDs) should be considered in the selection of materials, operative techniques and patient instructions related to prognosis and long-term cost-effectiveness.

**Objective:**

This study evaluated the long-term survival of fiber-reinforced, adhesively-bonded composite prostheses placed in posterior teeth.

**Material and Methods:**

Twenty-one patients that received adhesively bonded polyethylene inlay FPDs in posterior teeth were selected from a private practice dental office and invited to evaluation. Of the eligible 21 patients, 13 (mean age 50.3 ±11.5 years) agreed to be enrolled as participants, providing 22 restorations, as several subjects presented more than one inlay FPD. One dentist placed all inlay FPDs using Ribbond as reinforcement and Tetric Ceram/Durafil or Charisma/Renamel composite combinations, according to manufacturer’s instructions. Two independent calibrated operators performed the evaluation, using modified USPHS criteria. Survival functions of restorations were analyzed with Kaplan-Meier and Log Rank test (α = 0.05).

**Results:**

The majority of restorations received A or B scores. Four (18.2%) inlay FPDs fractured among the 22 evaluated. The mean estimate survival rate was 7 years (95% CI: 5.9 to 8.1), and the overall percentage of survival was 81.8%. There were no significant differences (p>0.05) between composite combinations or tooth location considering all clinical aspects evaluated and survival functions.

**Conclusion:**

Posterior fiber-reinforced fixed partial dentures exhibited acceptable clinical performance after a period up to 8 years.

## INTRODUCTION

Over the past decades, new developments in resin technology and patient demand for toothcolored restorations led to an increased use of resin-bonded fiber-reinforced fixed partial dentures (inlay FPDs) to replace a single missing tooth, as reported in several studies^1,4,13,24,31^. The use of ultrahigh molecular weight polyethylene (UHMWP) fibers is based on the improvement of the composite resin mechanical properties and behaviour^6,19,24^. This improvement depends on the fiber direction and pre-treatment. In order to reinforce the restoration in multiple directions, woven fiber and meshes have been proposed, where isotropic properties are achieved^17,24,30^. Incorporated into composite materials, the fibers provide enhanced fracture resistance, indicating their application even when high stress is present in the oral environment^10,12,25,28^. Although these constructions were originally made of metal combined with feldspathic ceramic, inlay FPDs are currently selected due to their various advantages when compared to metal-ceramic restorations, as a tooth-coloured material, presence of an adhesive and the tissue-saving properties of these constructions^36^.

Information on the longevity of inlay FPDs should be considered in the selection of materials, operative techniques and patient instructions related to prognosis and long-term cost-effectiveness. Despite the fact that survival rates considering other types of FPD framework are available^23^, few studies using fiber-reinforced composites in posterior teeth reported clinical performance or survival rates^5,14,35^. Moreover, as the majority of the studies report short-term evaluation periods^11,15,34^, there is limited information available on performance determinants and reasons of failure of inlay FPDs^3,8,14,27,29^. In fact, there appear to be few long-term clinical studies reporting survival rates of posterior polyethylene fiber-reinforced FPDs showing survival rates from 55 to 86%^2,3,16,20,21,33^. Therefore, evidence reporting clinical performance of inlay FPDs mainly from data generated in clinical practice is needed, especially as information on long-term survival is still scarce. The aims of this study were to report the survival rates of posterior fiber-reinforced composite restorations and compare the performance of two composite resin combinations used in inlay FPDs placed in a private dental practice. The follow-up period was up to 8 years (ranging from 12 to 96 months).

## MATERIALS AND METHODS

This study was a prospective, longitudinal evaluation, where the case reports of 21 adult patients were selected according to pre-determined inclusion criteria among the registers of a private practice dental office. These criteria included patients in continuous clinical follow-up considering success and failures in the last 8 years that had received polyethylene fiber-reinforced (Ribbond Co., Seattle, WA, USA) adhesively bonded composite resin 3-unit prostheses in posterior teeth. Resins used were Charisma (Heraeus-Kulzer, Wehrheim, Germany) together with Renamel (Cosmedent Inc., Chicago, IL, USA) or Tetric Ceram (Ivoclar Vivadent Inc., Schaan, Liechtenstein) together with Durafil (Heraeus-Kulzer); the resin combination for each inlay FPD was selected by chance. Patients presenting parafunctional habits, loss of occlusal stability or any medical condition that impaired correct hygiene procedures were excluded. Antagonist dentition was always in natural teeth.

Pontic areas larger than 7 mm were not considered for this treatment modality. The pontic was either a 2^nd^ premolar or 1^st^ molar (5-7 mm mesiodistal distance), while the abutments were the adjacent teeth. Thirteen of the 21 eligible patients (mean age 50.3±11.5 years) met the inclusion criteria and agreed to be enrolled as participants. All study subjects signed an informed consent form prior to the beginning of the clinical evaluation. This study was approved by the University Research ethics Committee. Although 13 patients were selected, 22 restorations were considered, as several patients presented more than one inlay FPD (Table 1).

**Tabela 1:** Description of the patients and inlay fiber-reinforced composite fixed partial dentures (FPDs)

**Gender**	**Material**	**Pontic space**	**n**	**Failures**
				
Female	Charisma	Lower Molar	1	1
n= 15 restorations	n=6	Lower Premolar	1	0
		Upper Premolar	4	2
	Tetric Ceram	Lower Molar	4	0
	n=9	Lower Premolar	1	0
		Upper Premolar	2	1
Male	Charisma	Upper Premolar	1	0
n= 7 restorations	n=1			
	Tetric Ceram	Lower Molar	1	0
	n=6	Upper Molar	1	0
		Upper Premolar	4	1
Total			22	4

**Figure 1:**
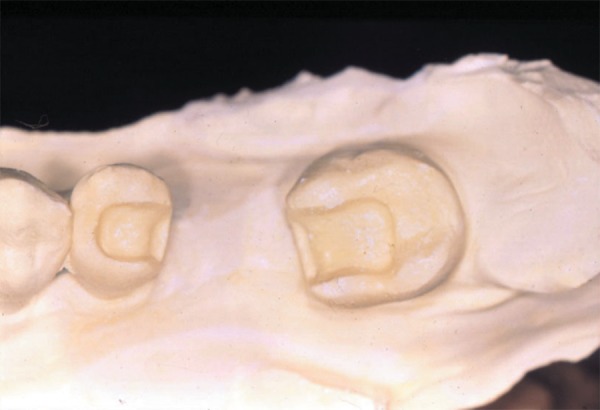
Cast obtained after cavity preparation, with conservative and expulsive shape, restricted to enamel margins, which provided an adequate restorative material thickness

One operator (PARR) carried out all clinical and laboratorial procedures. Preparations were performed using a 245-carbide bur (KG Sorensen, São Paulo, SP, Brazil) to remove previous restorations and a 4137 (KG Sorensen) diamond bur to achieve the cavity conservative and expulsive shape similar to those for inlay restorations as commonly described in literature^24^, without bevels in enamel. All cavities were restricted to enamel margins. Cavities had a minimum of 4 mm in depth and width in the occlusal box to accommodate composite and fibers and also to prevent fibers’ exposure. The proximal boxes were 6 to 8 mm deep and 4 mm wide (Figure 1).

Impressions were taken with a silicon rubber impression material (express, 3M eSPe, St. Paul, MN, USA) using a 2-step putty-wash impression technique and cavities were filled with a provisional methacrylate material (Fermit, Ivoclar Vivadent Inc., Schaan, Liechtenstein), followed by color selection performed using a shade guide system (VITAPAN Classical, VITA Zahnfabrik, Bad Säckingen, Germany).

**Figure 2:**
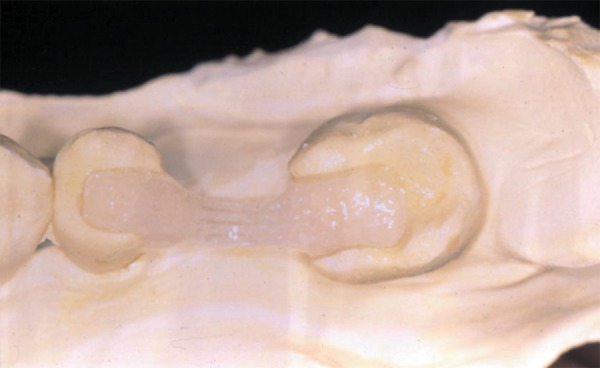
The polyethylene fiber was positioned in place with a layer of composite resin

**Figura 3:**
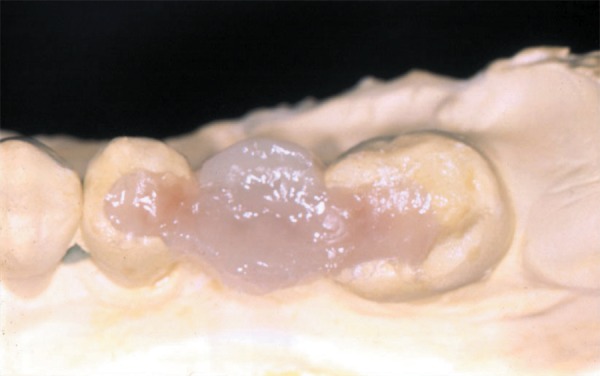
The fixed partial denture is completed with additional composite layers

**Figure 4:**
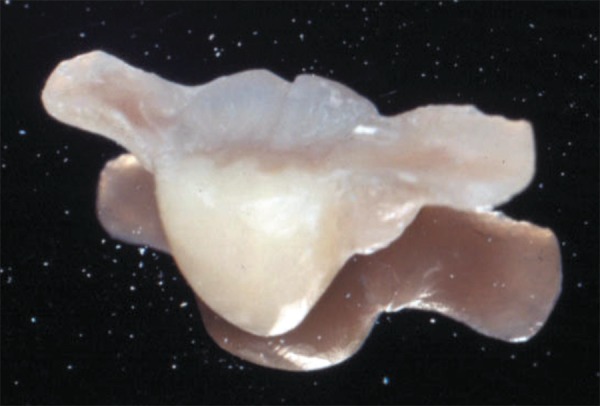
A closer view of the fiber-reinforced fixed partial denture after finishing and polishing procedures

**Figure 5:**
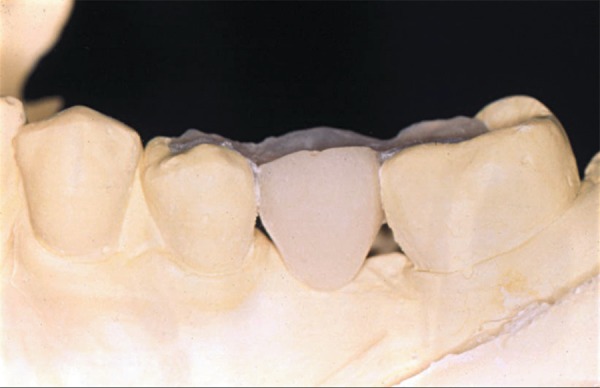
The finished restoration placed in the cast to test the adaptation

Casts obtained with type IV dental stone (Vigodent S.A. Indústria e Comércio, Rio de Janeiro, RJ, Brazil) were coated with cyanoacrylate (Super Bonder, Loctite, Brazil) prior to laboratory procedures. A 1 mm composite resin increment (Charisma or Tetric Ceram) was positioned in each pulpal-axial wall to retain the previously adhesive moistened (Scotch Bond, 3M ESPE) polyethylene fiber. The fiber was positioned with its extremities within the cavities on the non-polymerized composite, following pulpal and axial preparation contours. In the pontic region, the fiber was positioned 2 mm above gingival area. Next, composite and fiber were polymerized for 40 seconds with a light-curing unit operating at 600 mW cm^-2^ (Demetron LC Kerr, Orange CA, USA) (Figure 2). An additional 1 mm increment was placed and another polyethylene fiber was positioned in the same way as the previous one. Restorations were completed incrementally (in 2-mm-thick increments) and polymerized as described above. Gingival and buccal walls were completed with a microfilled composite resin (Durafil or Renamel) to ascertain translucence and surface smoothness^32^ (Figures 3). A closer view is demonstrating the restorations completed (Figure 4) and being tested in the model (Figure 5).

All enamel and cavosurface margins of preparations on abutments were acid-etched and coated with a bonding agent (Single Bond, 3M ESPE). Fiber-reinforced fixed partial dentures were treated with aluminum oxide (Micro-etcher, Danville Inc., Danville, USA), acid-etched, coated with Single Bond and luted with Rely X ARC cement (3M eSPe). Bonding agents and composites were placed according to manufacturer’s instructions, and FDPs were placed under rubber dam isolation and polymerized for 60 seconds on each aspect (occlusal, buccal, and lingual). Occlusal adjustments were carried out before cementation procedures. However, we re-checked occlusal contacts after cementation, as debonding may be related to improper occlusal adjustment in FPDs^34^.

**Figure 6:**
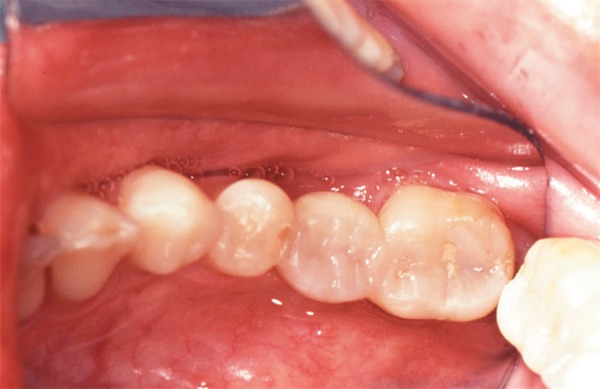
Occlusal view of the definitive restoration demonstrates esthetic integration reflecting harmonious integration of form and function

Finishing of the restorations was carried out before the luting procedure and completed immediately after placing. Cervical overhangs were removed with a # 12 scalpel blade and plastic finishing strips (3M ESPE). Proximal margins were finished with Sof-Lex XT discs (3M ESPE). The occlusal surfaces were finished with fine diamond finishing burs (KG Sorensen), multibladed carbide burs (Jet Burs), and polished with aluminum oxide points (Flexicups, Cosmedent Co., Chicago IL, USA) and a silicone brush (Jiffy Composite Polishing Brush, Ultradent South Jordan, UT, USA), with a polishing paste (enamelize, Cosmedent Inc., Chicago, IL, USA) (Figure 6). The same investigator that placed the restorations carried out baseline evaluations.

Direct evaluation was carried out with the modified USPHS criteria^9,26^. Indirect evaluation by standard photographs (Sony CyberShot DSC-F717, Sony electronics Inc., Tokyo, Japan) was used to complement data from direct evaluation.

Two calibrated examiners (MSC and TPC) worked independently to perform the evaluation, and an inter-examiner agreement of 80 *per cent* or more was obtained and considered statistically acceptable. evaluation was blind in relation to the examiners. Radiographic examination was carried out when necessary to complement the clinical evaluation. Additionally, all patients had a complete annual periapical radiographic exam, which was examined by examiners.

Descriptive statistics were used to describe the frequency distributions of the evaluated criteria. Differences between material combinations were analyzed with Chi-square test and differences between baseline and 96-month evaluations were analyzed with McNemar’s test (α=0.05). For each FPD evaluated, the time to failure or longevity in months was recorded and the survival of the restorations or subsets of restorations grouped on the basis of variables (material and tooth location) were displayed using Kaplan-Meier survival curves^7,18^. Comparison between survival curves was determined with the Log Rank test.

## RESULTS

Thirteen patients (61.54% female and 38.46% male, mean age 50.31±11.48) agreed to participate in the study. No statistically significant difference was found between female and male concerning failure rates (Table 1). Of the 15 Tetric Ceram + Durafil restorations evaluated, 1 was replaced during the clinical service, and 14 restorations remained without any additional treatment. Of the 7 Charisma + Renamel restorations, 3 were replaced, with 4 restorations remaining *in loco*. All 4 failures recorded were fracture-related, and fractures always occurred in the abutment-pontic junctions.

**Figure 7:**
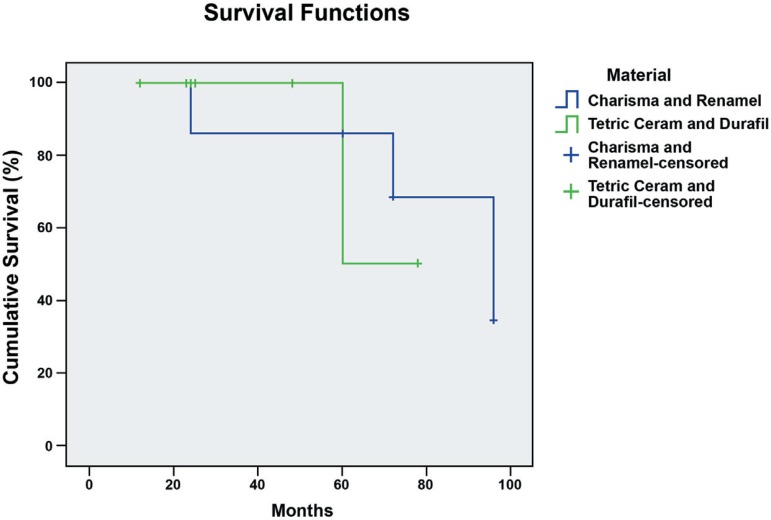
Survival functions of fiber-reinforced fixed partial dentures according to composites combinations. Differences among curves were not statistically significant by Log Rank Test (p=0.98)

**Figure 8:**
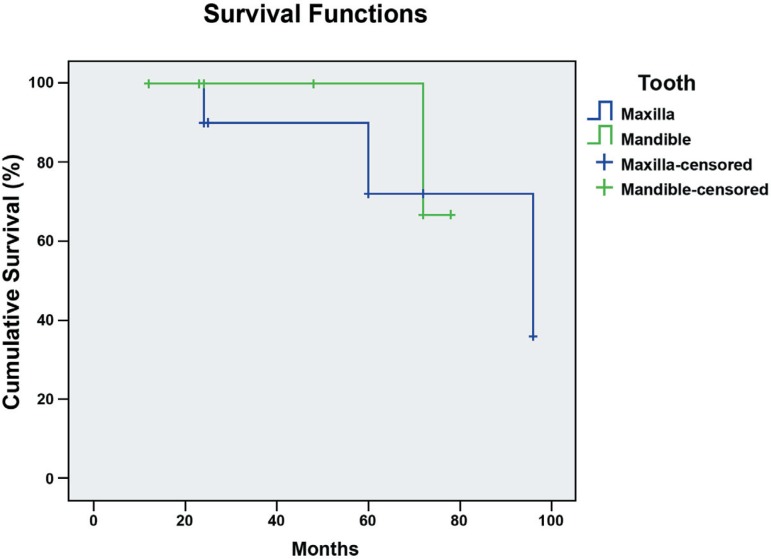
Survival functions of fiber-reinforced fixed partial dentures according to tooth location. Differences among curves were not statistically significant by Log Rank Test (p=0.77)

The majority of the restorations evaluated exhibited score A in all of the evaluated criteria (Table 2). Neither material nor time of evaluation demonstrated to be significant factors regarding any clinical aspect directly evaluated (p>0.05). No case of secondary caries was found for both materials (Table 2). Kaplan Meier overall survival probability at 8 years was 34.2% (mean estimate survival of 7 years) (Tables 3 and 4). There were no statistically significant differences (p>0.05) between survival functions to composites combinations or teeth location by the Log Rank Test (Figures 7 and 8).

**Tabela 2:** Results of direct assessment for the fiber-reinforced composite fixed partial dentures (FPDs) that remained in place after the evaluation period

**Criteria**		**Restorative Material**			
	**Charisma + Renamel**			**Tetric Ceram + Durafil**	
		**Baseline**	**Up to 96-month**	**Baseline**	**Up to 96-month**
					
Color Match	A	7	4	15	14
	B	0	0	0	0
	C	0	0	0	0
Marginal Adaptation	A	7	3	15	11
	B	0	1	0	3
	C	0	0	0	0
Anatomic Form	A	7	3	15	13
	B	0	1	0	1
	C	0	0	0	0
Surface Roughness	A	7	4	15	14
	B	0	0	0	0
	C	0	0	0	0
Marginal Staining	A	7	2	15	9
	B	0	2	0	5
	C	0	0	0	0
Occlusal Contacts	A	7	3	15	14
	B	0	1	0	0
	C	0	0	0	0
Sensitivity	A	7	4	15	14
	B	0	0	0	0
	C	0	0	0	0
Secondary Caries	A	7	4	15	14
	B	0	0	0	0
Inlay FPDs Retention	A	7	4	15	14
	B	0	0	0	0

No statistically significant differences were found between materials (qui square test) or between baseline and final evaluation (McNemar test) considering any clinical aspect evaluated (p>0.05). Scores A and B were considered as success (except for secondary caries and inlay FPDs retention) for statistical analysis.

**Tabela 3:** Means for survival time in months according to material

**Material**	**Mean**			
			**95% Confidence Interval**	
	**Estimate**	**Standard Error**	**Lower Bound**	**Upper Bound**
				
Charisma / Renamel	81.00	11.78	58.51	104.69
Tetric Ceram / Durafil	69.00	6.36	56.53	81.47
Overall	84.00	6.95	70.37	97.63

**Tabela 4:** Means for survival time in months according to tooth location

**Tooth Location**	**Mean**			
			**95% Confidence Interval**	
	**Estimate**	**Standard Error**	**Lower Bound**	**Upper Bound**
				
Maxilla	82.32	10.35	62.04	102.60
Mandible	76.00	1.63	72.80	79.20
Overall	84.00	6.95	70.37	97.63

## DISCUSSION

Longitudinal, prospective studies and retrospective analyses of dental records are the only feasible tools to use in evaluating the longterm performance of restorations *in vivo* . This study was an up to 8 years longitudinal retrospective clinical-follow up evaluating the performance of adhesively bonded fiber-reinforced composite fixed partial dentures.

The success rate of first-generation, multiphase polymer matrix fiber-reinforced composite FDPs including surface-inlay- and hybrid- retained designs and consisting of 1 to 3 pontics was 93% after a maximum of two years follow-up^34^. Other studies have also reported high survival rates (100% at 12, 15 and 24 months) for 3-unit inlay retained fiber-reinforced composites FDPs with short-term follow-up^2,11,15^. Clinical performance of surface-retained adhesive composite fixed partial dentures reinforced by an UHMWP has also reported a 91.3% survival at the end of two years and 78.3% after a maximum of 3 years^33^. In the present study, the overall estimate survival mean was of 84 months (7 years) for the restorations in the present study, and the overall percentage of survival was 81.8%. Pröbster and Henrich^23^ (1997) reported 61% of overall survival rates and 76% of functional survival rates for metal framework fixed partial dentures after an 11-year period of evaluation, while reported significantly higher overall and functional survival rates after 63 months, 75% and 93%, respectively in fiber-reinforced ones. Although it is not possible to directly compare metal-ceramic to fiber-reinforced FPDs with regard to their mechanical properties, when considering their survival, they should be compared considering their cost, less time-consuming procedure, material color, presence of an adhesive and tissue-saving properties^36^. The lower survival rates found in the present study can be attributed to the large range (12 to 96 months) of clinical service time reported. Yet, success rates might also be affected by a long-term assessment, *i.e.*, inlay FPDs evaluated after a delivery period of two years or more may exhibit a twice-higher failure risk^8^. Additionally, fiber-reinforced composite restorations may present different mechanical properties compared to cast alloys and the differences related to the adhesive properties may influence the survival rates. However, the estimate mean survival was higher than the 55.03 months described by Vallittu^35^ (2004), which is associated to the longer evaluation period reported in the present study.

Longevity of fixed partial dentures fiberreinforced composite restorations is dependent upon many different factors, including material, maxillary or mandibular arches, patient- and dentist-related. Moreover, patient factors such as oral hygiene, dietary habits, preventive measures, fluoride availability, compliance in recall and cooperation during treatment, and oral environment are relevant topics when considering the restoration durability^21^. Although the evidence that inlay FPDs placed in the mandible show a higher failure risk than those placed in the maxilla^8^, in the present study maxillary and mandibular restorations did not differ in survivals, in agreement with the previous findings reported by Vallittu^35^ (2004). However, further clinical investigations are still needed for improved long-term clinical performance, as clinical trials with larger number of inlay FPDs could confirm or decline differences between arches. The main reason for failure in the present study was fracture, whilst neither secondary caries nor postoperative sensitivity was related. It is important to highlight that the clinical environment where the inlay FPDs were placed has a dental practice focused on health promotion, with a preventive approach and based on the control of caries disease. In addition, patients included in this study had regularly attended the dental office with at least one appointment *per* year.

Fiber-reinforced partial dentures fracture strength depends on several factors including the elastic modulus of the supporting substructure, the preparation design, occlusal load of the span and the characteristics of the manufacturing and laboratory process, and the materials used to fabricate the prosthesis^24^. The failures recorded in the present study could somewhat be attributed to cavity preparation deficiency and/or excessive occlusal load as result of a slightly larger interabutment distance. In addition, clinical trials have determined that larger prosthetic spaces especially in mandible are a potential risk factor for posterior inlay FPDs^8^, and therefore should be avoided. All fractured inlay FPDs were replaced after cavity re-contouring, and no immediate evidence of new failures was recorded subsequently, emphasizing the importance of adequate cavity preparation in order to provide adequate fracture strength to inlay FPDs. Conversely, few studies have focused on cavity preparation for inlay FPDs, and the principles governing standard cavity preparation have not been well established^24,27^. When making box preparations for an inlay FPD, if pre-existing restorations are present, they can determine abutment shape. Otherwise, when teeth are intact, mechanical and biological aspects must be considered in choosing the preparation design: the occlusal box should be sufficiently deep to accommodate the fiber and a protective layer of composite; and the proximal box should be as deep as possible in the gingival direction to ensure an adequate amount of fiber and composite, and to provide maximal strength in the connection area. Also, the margins must be located within the enamel for better long-term marginal adaptation^24,27^, and to assure the correct dental biofilm control and avoid tissues diseases.

The reduction in sound dental structure removal, the bonding capacity - preventing microleakage and reinforcing the remnant dental structure when compared to other framework materials and the esthetics are some of the reasons for the increasing use of fiber-reinforced composite fixed partial dentures placement. The results of this investigation suggest that fiber-reinforced fixed partial dentures may be a feasible choice for a long-term provisional treatment for a single tooth replacement. Despite the small number of patients in this study, the results were obtained following a standardized protocol, with a single operator placing the restorations, and using only two different combinations of composites. Information on clinical survival of fiber-reinforced fixed partial dentures lacks in literature^22^, and hence the findings of the present study may help bringing into discussion some important aspects related to the performance of these restorations, with some interesting clinical observations. However, a larger number of evaluated restorations and longer evaluation periods in multicenter studies could be more appropriate in order to generate stronger scientific evidence.

## CONCLUSION

The results of this study suggest that clinical performance of posterior fiber-reinforced fixed partial dentures evaluated was acceptable after a period of up to 8 years, and that inlay FPDs may be a feasible alternative for the replacement of a single missing tooth.
